# NRTPredictor: identifying rice root cell state in single-cell RNA-seq via ensemble learning

**DOI:** 10.1186/s13007-023-01092-0

**Published:** 2023-11-04

**Authors:** Hao Wang, Yu-Nan Lin, Shen Yan, Jing-Peng Hong, Jia-Rui Tan, Yan-Qing Chen, Yong-Sheng Cao, Wei Fang

**Affiliations:** The Innovation Team of Crop Germplasm Resources Preservation and Information, Institute of Crop Sciences, Chinese Academy of Agricultural Sciences, Beijing, 100081 China

**Keywords:** Machine learning, Marker genes, scRNA-seq, Rice root tips, Cell subpopulations

## Abstract

**Background:**

Single-cell RNA sequencing (scRNA-seq) measurements of gene expression show great promise for studying the cellular heterogeneity of rice roots. How precisely annotating cell identity is a major unresolved problem in plant scRNA-seq analysis due to the inherent high dimensionality and sparsity.

**Results:**

To address this challenge, we present NRTPredictor, an ensemble-learning system, to predict rice root cell stage and mine biomarkers through complete model interpretability. The performance of NRTPredictor was evaluated using a test dataset, with 98.01% accuracy and 95.45% recall. With the power of interpretability provided by NRTPredictor, our model recognizes 110 marker genes partially involved in phenylpropanoid biosynthesis. Expression patterns of rice root could be mapped by the above-mentioned candidate genes, showing the superiority of NRTPredictor. Integrated analysis of scRNA and bulk RNA-seq data revealed aberrant expression of Epidermis cell subpopulations in flooding, Pi, and salt stresses.

**Conclusion:**

Taken together, our results demonstrate that NRTPredictor is a useful tool for automated prediction of rice root cell stage and provides a valuable resource for deciphering the rice root cellular heterogeneity and the molecular mechanisms of flooding, Pi, and salt stresses. Based on the proposed model, a free webserver has been established, which is available at https://www.cgris.net/nrtp.

**Supplementary Information:**

The online version contains supplementary material available at 10.1186/s13007-023-01092-0.

## Background

Rice (*Oryza sativa L.*) is one of the most important food crops in the world, supporting as a staple food for more than half of the global population [1, 2]. The increase in rice production will have a significant impact on world food security, making it necessary to explore new strategies to improve rice yield [3]. The roots are fundamentally important for plant growth and development, anchoring the plant to its growth substrate, facilitating water and nutrient uptake from the soil, and promoting continuous rice yield increase [4–6]. Understanding the cell heterogeneity and gene regulatory networks of rice root development is a frontier field for improving its productivity [7, 8].

Single-cell RNA-seq (scRNA-seq) is gradually being used in plants to mine heterogeneity between tissue types and within cells, thus providing a more accurate and integrated understanding of their role in the life process [9–11]. For example, Liu et al. [12] reported the single-cell transcriptome from the root tip of rice, identifying most of the major cell type transcriptional landscape of rice roots at the single-cell resolution. Denyer et al. [13] used High-throughput scRNA-seq to demonstrate the expression atlas of *Arabidopsis* roots, capturing its precise spatiotemporal information and revealing key regulators across all major cell types. With the availability of scRNA-seq data, cell type identification is an important step towards various downstream analysis [14–16]. In some cases, we lack good markers of crucial cell populations in defining cell types. Training an effective machine learning prediction model to mine molecular markers and identify cell subpopulations based on existing single-cell datasets is a time-saving and labor-saving approach [17].

**Fig. 1:**
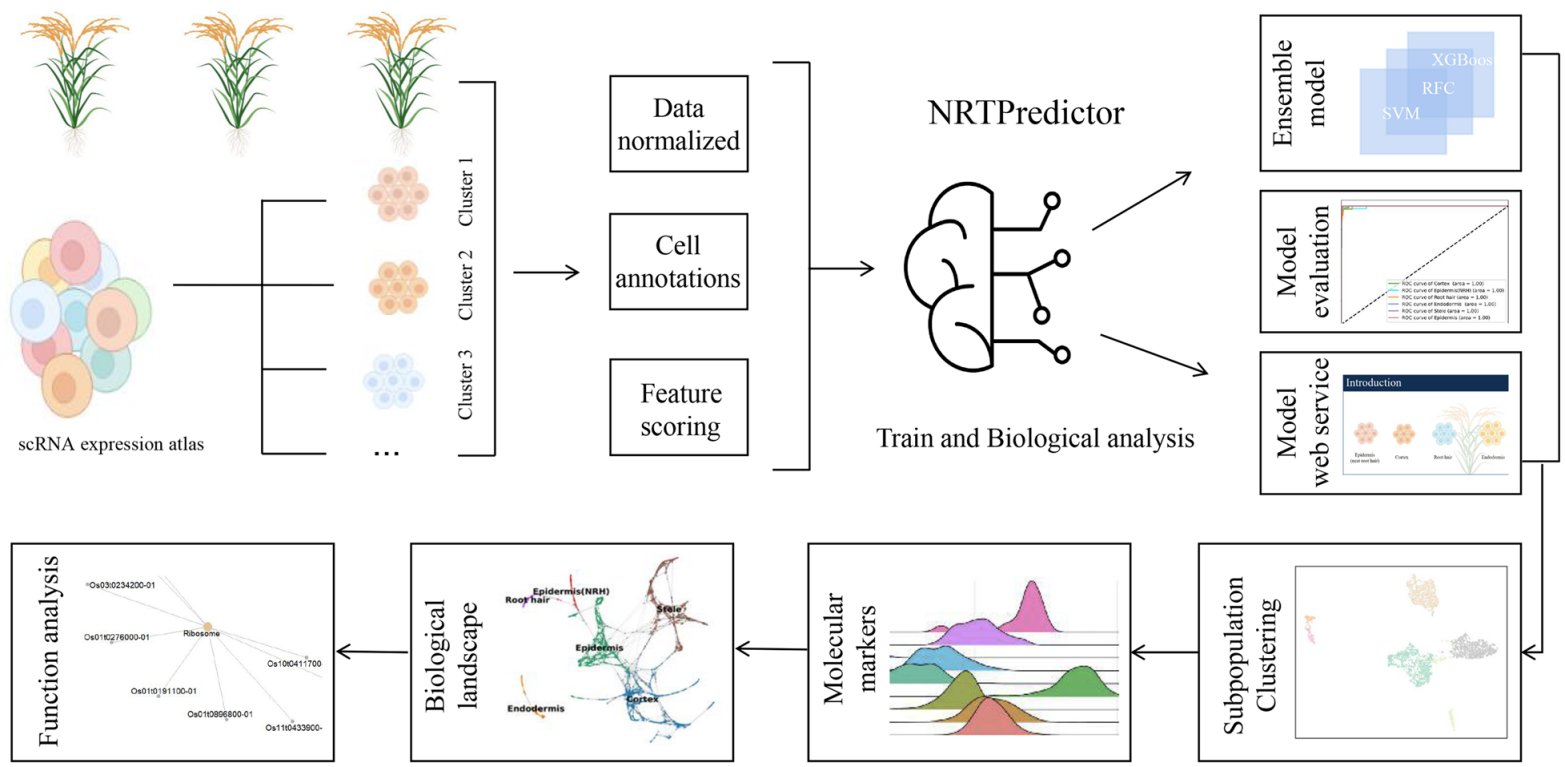
The workflow of constructing NRTPredictor

To address the above limitations, we proposed an ensemble computing framework, named NRTPredictor, which enabled the model to capture cell subpopulation biomarkers of Nipponbare root tips and predict stages of cells (Fig. 1 and Additional file 1: Figure S1). NRTPredictor integrated recent popular three feature selection methods (MIC, F-score and CV2) and four machine learning models (SVM, XGBoost, Lightgbm and RFC) to evaluate the importance of genes on cell subpopulations of Nipponbare root tips prediction.

Moreover, we successfully applied our NRTPredictor model on data unseen during training and demonstrated its superior predictive performance. By performing biological analysis of the optimal genes, we detailed potential marker genes, which could help biologists better understand the heterogeneity of Nipponbare root tips. In addition, we integrated rice root single-cell marker genes with RNA-seq data of flooding, salt, and pi stresses and found that Epidermis cell subpopulations may play critical roles in rice stress mechanisms. Our work provides a comprehensive understanding of machine learning to mine marker genes at the single-cell level in rice and enhances the understanding of stress physiological processes, which provides insights for improving current rice stress tolerance strategies.

## Results

### Identification of significant genes using feature selection and machine learning

**Fig. 2:**
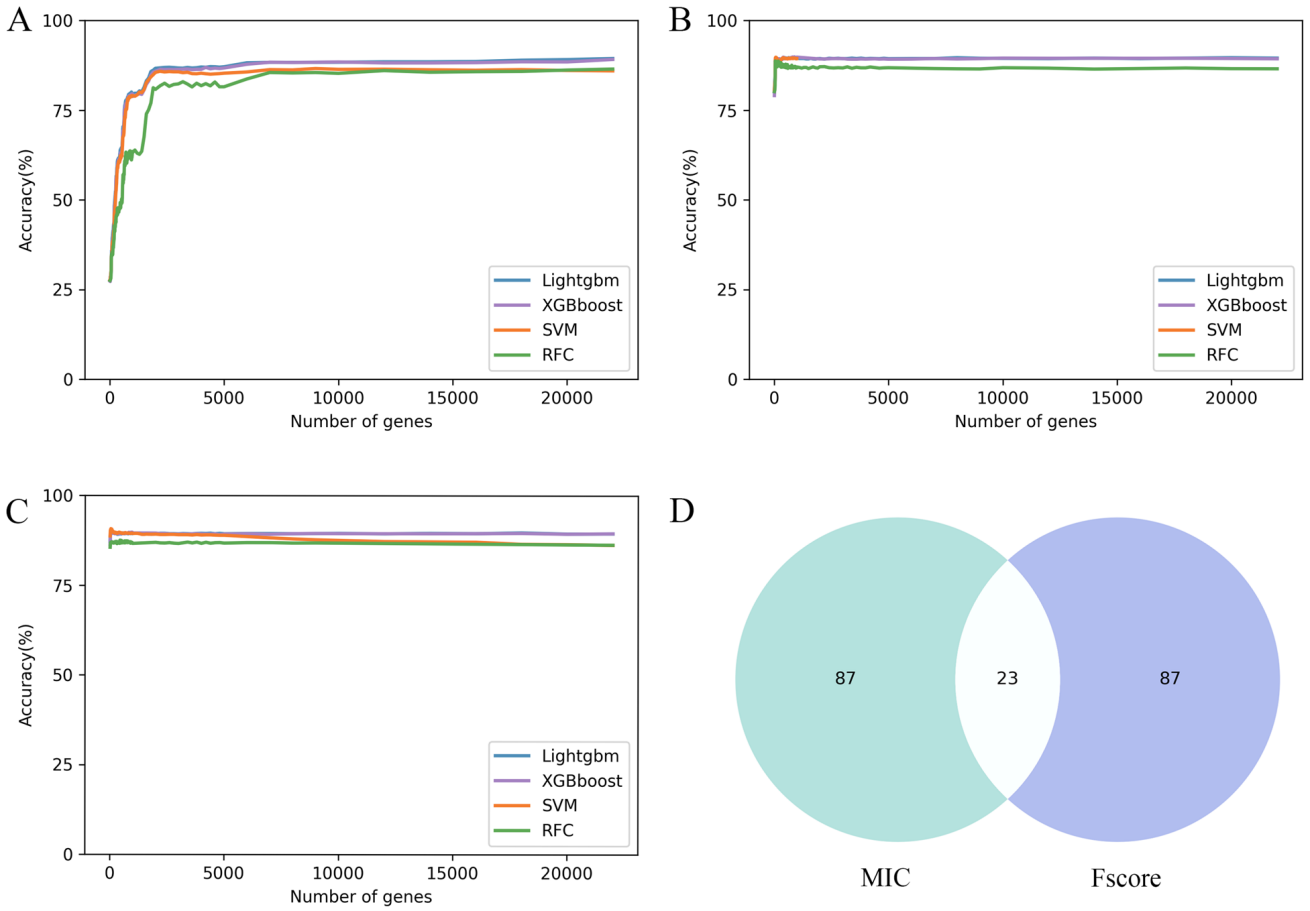
The results of feature selection. **A**, **B** and **C** The IFS curves show the performance of three feature selections (CV2, F-score and MIC) and the four classifiers in different gene subsets. **D** Comparative Venn diagram of the top 110 genes in MIC and F-score

For identifying the significant genes related to subpopulations of Nipponbare root tip cells, we used three feature selection methods (MIC, CV2 and F-score) to evaluate the importance of the 39,219 genes and ranked them according to their contribution value. Genes with importance score less than or equal to zero were excluded. The MIC, CV2, and F-score extracted 23,157 genes respectively. Next, the machine learning models combined with incremental feature selection (IFS) were used to determine the optimal gene subsets. (Fig. 2A, B, and C). Based on five-fold cross-validation, single-cell gene expression matrices (Normalization of raw read count) were used as input features to train machine learning models (SVM, RFC, XGBoost and Lightgbm).

The train dataset results showed that MIC combined with SVM (MIC_SVM) achieved optimal prediction performance using top 110 genes, with the accuracy of 97.23% (Table 1). It is worth noting that the four machine learning models combined with the MIC and F-score also obtained superior prediction performance. To avoid the MIC and F-score having the same gene preference, we selected the top 110 genes in the score ranking of the two feature selection methods for comparison. As observed from Fig. 2D, MIC and F-score have few intersections and sufficiently differences. Using the 110 optimal genes on test data, MIC_SVM also predicted the best performance, with accuracy, precision, recall, and F1-measure of 96.72%, 95.15%, 94.84 and 94.92%, respectively (Table 2).

**Table 1:** Performance evaluation of different feature selection combined with machine learning schemes (Train dataset)

Method	Feature selection	No. of features	Accuracy %
Lightgbm	F-score	150	96.53
XGBoost	F-score	430	97.88
SVM	F-score	180	96.34
RFC	F-score	210	94.41
Lightgbm	CV2	20,000	97.11
XGBoost	CV2	20,000	97.59
SVM	CV2	7000	94.22
RFC	CV2	14,000	89.71
Lightgbm	MIC	100	95.49
XGBoost	MIC	100	96.59
SVM	MIC	110	97.23
RFC	MIC	120	93.55

**Table 2:** Performance comparison between NRTPredictor and the other algorithms (Test dataset)

Method	Feature selection	No. of features	Accuracy %	Precision %	Recall %	F1-measure %
Lightgbm	F-score	150	96.53	94.27	93.37	93.78
XGBoost	F-score	430	97.88	96.48	96.33	96.39
SVM	F-score	180	96.34	94.02	93.35	93.66
RFC	F-score	210	94.41	93.09	85.51	88.14
Lightgbm	CV2	20,000	97.11	95.48	94.49	94.97
XGBoost	CV2	20,000	97.59	96.27	95.56	95.89
SVM	CV2	7000	94.22	93.45	89.92	91.27
RFC	CV2	14,000	89.71	88.02	85.43	85.37
Lightgbm	MIC	100	95.49	94.49	93.70	94.02
XGBoost	MIC	100	96.59	94.80	94.56	94.61
SVM	MIC	110	96.72	95.15	94.84	94.92
RFC	MIC	120	93.55	91.18	81.17	85.18
NRTPredictor	MIC	110	98.01	95.63	95.45	95.95

### NRTPredictor construction and performance in validated datasets

**Fig. 3:**
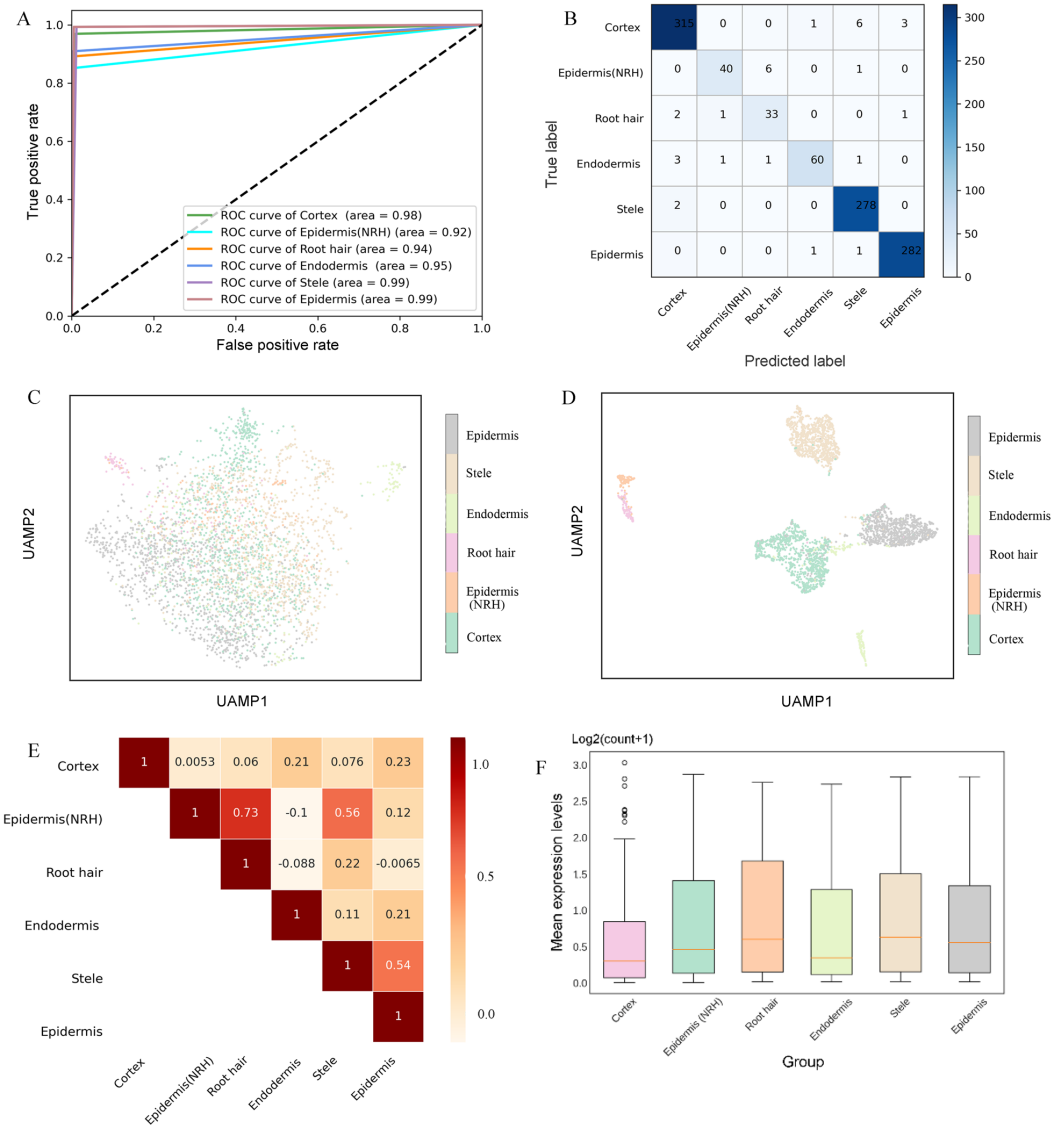
Predictive performance of NRTPredictor. **A** ROC curves for NRTPredictor on training set. **B** The confusion matrix shows the accuracy of NRTPredictor using 110 genes from MIC_SVM algorithm on test dataset. **C** and **D** The clustering effect on 3463 cells was evaluated using 110 marker genes and all genes (**C** represents all genes, **D** represents the 110 marker genes). Each point represents a sample in the dataset, and different categories of samples are given different colors. **E** Correlation analysis of six rice root cell subpopulations at the level of 110 marker genes. **F** Boxplots of Mean expression levels (Normalization of raw read count) of 110 marker genes in six rice root cell subpopulations

To further improve the performance of the model, we integrated the above four basic classifiers (SVM, RFC, Lightgbm and XGBoost) based on different weight assignments, called NRTPredictor. Ensemble models outperform individual models, with accuracy, precision, recall, and F1-measure of 98.01%, 95.63%, 95.45, and 95.95%, respectively. The receiver operating characteristic (ROC) curve and confusion matrix further verified the prediction performance of the NRTPredictor in six rice root cell subpopulations, and the low misclassification rate proved the demonstrated power of the NRTPredictor (Fig. 3A and B). To explore the scalability of the model, we trained NRTPredictor on the *Arabidopsis* dataset, and the results showed that NRTPredictor still had optimal prediction performance in both the training and test sets (Additional file 2: Table S3).

In addition, we have carried out a performance comparison between the pseudobulk differential expression analysis and our proposed NRTPredictor method. Notably, the pseudobulk analysis identified 1216 genes (Additional file 2: Table S4) overlapping with 98 of the 110 genes mined by our method (Additional file 1: Figure S2). However, ensemble model exhibited superior predictive performance when using the 110 genes (Tables 1 and 2). Thus, while the pseudobulk analysis may identify more differentially expressed genes, the 110 genes identified by our method more accurately represented cell subpopulations with less computational complexity.

### Investigating NRTPredictor model interpretability

**Fig. 4:**
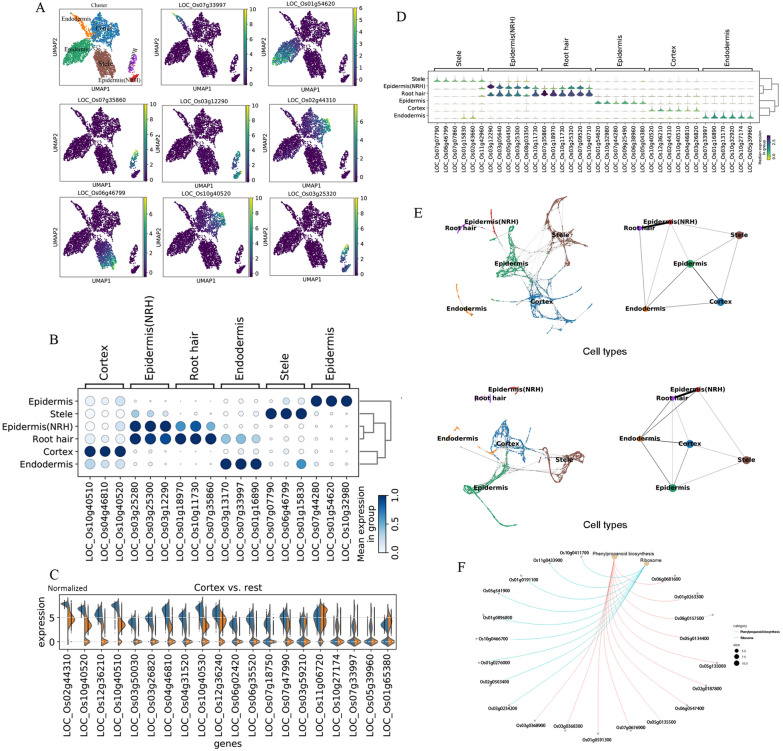
Computational analysis of 110 marker genes. **A** UMAP shows potential marker genes for rice root cell fate determination. **B** The marker genes of rice root cell subpopulations have been reported. **C** Comparison of marker genes selected by MIC_SVM (110 marker genes) using split violin plots. The expression level of marker genes in specific cells is shown on the left (Blue), and the total expression level in the remaining five cell types is shown on the right (Orange). **D** High expression marker genes screened by Scanpy. **E** Expression trajectory analysis of 110 marker genes (downward) and all genomes (upward) of rice root cell subpopulations colored by cell type using PAGA. The thicker the line, the closer the cell connection. **F** KEGG enrichment analysis of 110 marker genes

To explain the performance of the proposed model, NRTPredictor gene set (110 marker genes) was extracted and visualized. The Uniform Manifold Approximation and Projection (UMAP) of 3,463 single cells indicated that the overall performance of the 110 marker genes was significantly better than all genes (Fig. 3C and D). Specifically, samples from different categories appeared almost intermixed during the clustering process utilizing all genes (Fig. 3C). However, using the 110 optimal genes yields a clear distribution of cell subpopulations, showing favorable clustering results (Fig. 3D). We also performed a correlation analysis of 110 marker genes, and the same subpopulation of cells showed strong correlation (Fig. 3E). These genes can be used to classify subpopulations of rice root tip cells. Moreover, by analyzing the expression of 110 genes in six cell subpopulations, high expression was found in Stele, Root_hair, and Epidermis, and low expression was found in Cortex (Fig. 3F). Accurate capture of genes involved in lineage identification helps to understand the cell subpopulations in rice root tips. We observed that LOC_Os02g44310 and LOC_Os10g40520 were specifically expressed in Cortex. LOC_Os07g33997, LOC_Os01g64520 and LOC_Os06g46799 respectively exhibited high specificity and expression in Endodermis, Epidermis and Stele, while LOC_Os07g35860 and LOC_Os03g25320 were highly expressed in Epidermis (NRH) (Fig. 4A, Additional file 1: Figure S3, and Additional file 2: Table S5). These highly ranked genes can be used as biomarkers to identify subpopulations of rice root cells and provide some support for further biological findings (Additional file 2: Table S5). In addition, we also successfully captured some reported cell subpopulation marker genes (Fig. 4B), such as LOC_Os03g25280, LOC_Os01g18970, and LOC_Os07g44280 [12, 18].

### Expression analysis of NRTPredictor gene set

Further, we explored the representative capacity of 110 marker genes in the biological landscape. We utilized Scanpy to compare gene expression levels between the top 20 genes within each cell subpopulation and their expression levels across the remaining five clusters. For example, the expression levels of LOC_Os12g36210, LOC_Os3g26820, and LOC_Os04g31520 in the Cortex cell subpopulation were higher than their sum in the remaining five cell subpopulations, respectively (Fig. 4C). For the Epidermis, LOC_Os10g32980, LOC_Os01g54620 and LOC_Os07g44280 show high levels of expression and they can be potential marker genes (Additional file 1: Figure S4). The results demonstrated that NRTPredictor had irreplaceable advantages in processing scRNA-seq data and does not rely on a priori biological background. Using multiple genes to characterize cell subpopulations of rice root tip could have greater ability to mark. We showed the top six specific genes with the highest expression in each cell subpopulation. As shown in Fig. 4D, when the co-expression of LOC_Os07g07790, LOC_Os06g46799, LOC_Os07g07860, and LOC_Os01g15830 is observed at high expression levels within a specific cell, that cell can be identified as a Stele cell.

Single-cell expression profiles containing all genes and 110 genes, respectively, were used as input to construct partition-based graph abstraction (PAGA) to describe the biological landscape (Fig. 4E). On the graph, the same topological structure was shown, such as the strong connections between Epidermis (NRH) and Root hair, suggesting that NRTPredictor screened for key molecular markers and removed redundant information (Fig. 4E). We performed Kyoto Encyclopedia of Genes and Genomes (KEGG) enrichment analysis on 110 genes to explore their functions in key biological pathways and processes. (Fig. 4F and Additional file 2: Table S6). The results exhibited that a large number of genes were enriched in the phenylpropanoid biosynthesis pathway, suggesting that these genes may be involved in the regulation of lignin and flavonoid synthesis, which played critical roles in plant growth and development, abiotic and biotic stresses [19–21].

Related studies reveal that proteins encoded by *OsCAD2* (LOC_Os02g09490, Os02g0187800) play a role in monolignol biosynthesis [22]. The expression of *OsCAD2* was most tightly associated with the transcription of genes related to lignin biosynthesis, indicating that *OsCAD2* is primarily responsible for monocotyledonous lignin biosynthesis in rice [23]. In addition, caffeic acid O-methyltransferase (*COMT*, LOC_Os08g06100, Os08g0157500), encoded in sorghum, has been shown to be one of the key enzymes in monolignol biosynthesis [24]. Result showed that LOC_Os02g09490 (Os02g0187800) and LOC_Os08g06100 (Os08g0157500) are specifically expressed in Epidermis cells (Additional file 2: Table S4 and S7), which was closely related to the protective function of root tip Epidermis cells in soil.

### Multi-omics data integration of scRNA-seq and Bulk RNA-seq

To simultaneously define expression changes at the global and cellular levels, we also performed bulk RNA-seq analysis on rice root cells under stress and control samples in parallel. The PPRD database has curated a substantial collection of publicly available rice RNA-seq data, enabling users to query the expression levels of genes in various tissues, developmental stages, abiotic and biotic stresses conditions [25]. Based on the PPRD database, we revealed that all 12 genes enriched in the phenylpropanoid biosynthesis pathway were expressed at high levels in the root, demonstrating that the key core genes were screened (Additional file 1: Figure S5). We further investigated the expression profiles of these 12 genes under stress conditions by querying PPRD database, and found their aberrant expression under salt, pi, and flooding stress (Additional file 1: Figure S6). In addition, six of these genes were expressed at high levels in Epidermis cell subpopulations, suggesting that Epidermis cells play a major role in regulation under stress conditions (Additional file 1: Figure S6). We then focused on the bulk RNA-seq-specific expression patterns related to salt stress, and the results showed that Epidermis cell subpopulations have a positive role in studying the molecular mechanism of salt stress in rice (Additional file 1: Figure S7).

### Webserver implementation

**Fig. 5:**
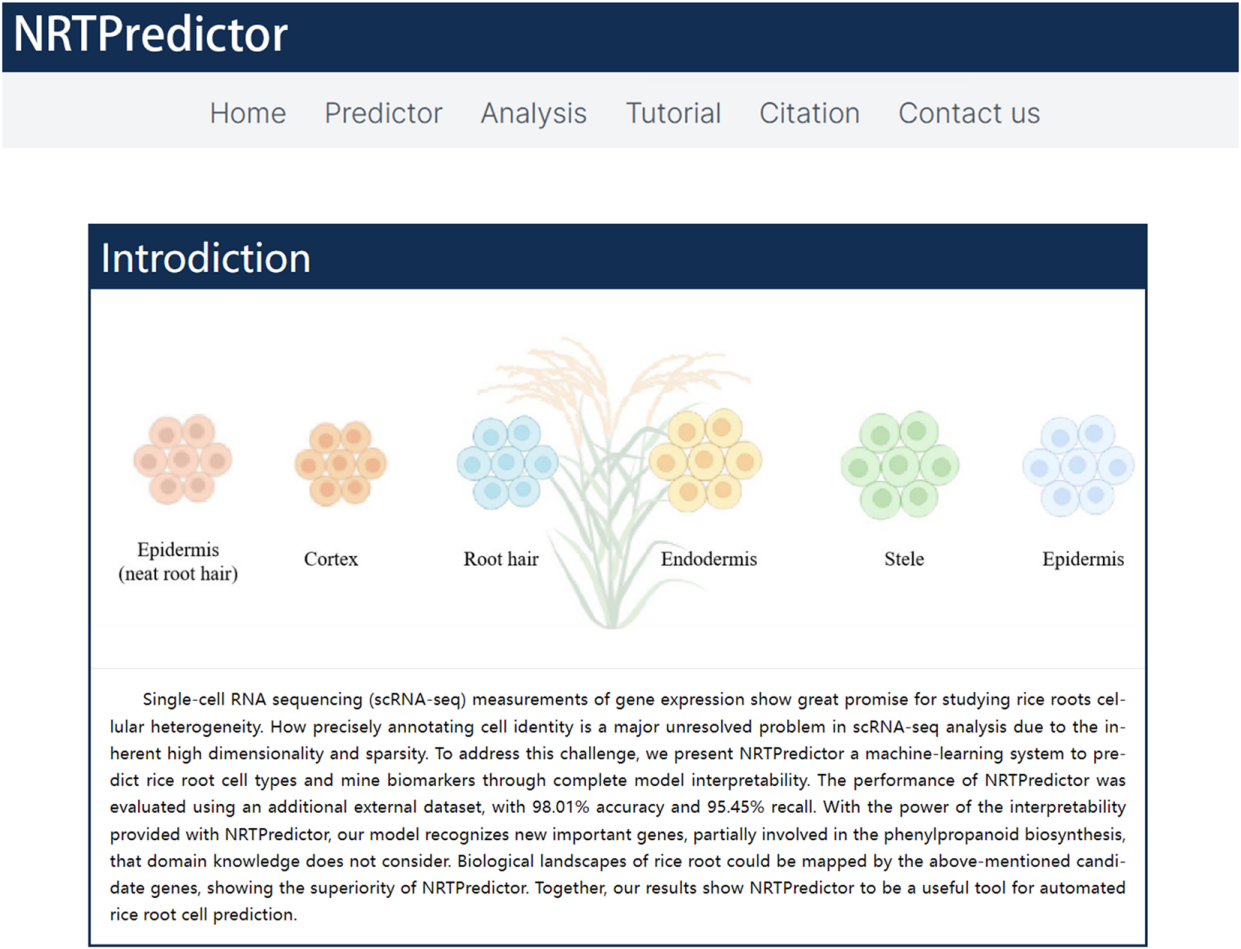
NRTPredictor model web server platform

By this research, the NRTPredictor webserver has been established and is freely available at https://www.cgris.net/nrtp. The home user interface of NRTPredictor was shown in Fig. 5. Click on the “Predictor” button to enter the service module. Researchers can submit simple CSV file with gene expression matrix as the input. Click on the “Submit”, the NRTPredictor webserver will process the submitted tasks, predict and return result file. We also provided the example file and step-by-step guide for users, which can be seen in the ‘Tutorial’ module of web service.

## Conclusion

A long-standing problem in scRNA-seq analysis of rice roots is that there are very few marker genes for cell types [26, 27]. Moreover, manual assignment of rice root cell types can result in high variability of cell annotation between research groups and poor reproducibility in cell identification between experiments [27, 28].

In this study we presented NRTPredictor, an expression atlas-based ensemble learning framework for modeling scRNA-seq data. NRTPredictor, which uses three feature selection methods and four machine learning algorithms to access global gene expression patterns and molecular events in rice root cells, is the first study to combine single-cell rice data with artificial intelligence. Experimental results on test datasets and cluster analysis demonstrated the effectiveness of our proposed NRTPredictor, which allowed researchers to perform automatic annotation of cells of interest. With NRTPredictor, we also identified a set of genes that could be robust cell-type markers for subpopulations of rice root tip cells. Visualization of the expression patterns of the optimal gene set showed that the optimal gene set retained the main patterns of the original biology and has great potential to annotate rice scRNA-seq datasets. We integrated the scRNA-seq and bulk RNA-seq to reveal that Epidermis cell subpopulations play a central role in rice response to flooding, salt and pi stress.

While this study provides valuable insights, it is important to acknowledge its limitations. One major constraint is the small sample size and the lack of external datasets to validate the model. The collaborative effort in data collection may facilitate improving the model. Despite this potential limitation of the current study, our work provides a resource to study the physiological functions of rice root cell types at the molecular level and at single-cell resolution and to reveal the unique molecular events that drive the development of resistant cells in rice. We hope that NRTPredictor will be a powerful bioinformatics tool providing insight into the genetic basis of cell fate decisions in rice roots, which is indispensable for interpreting cell-specific functions.

## Methods and materials

### Dataset construction and Preprocessing

Single-cell transcriptome data from root tips of Nipponbare containing 3463 cells were collected from the National Center for Biotechnology Information (GSE146035) [12]. The dataset covers specific rice root cell subpopulations of interest and is easily accessible, ensuring transparency and verifiability. In addition, the dataset has a moderate sample size and high-quality sequencing data, and was therefore selected for our analysis. Based on the same processing method used by Liu et al. [12], the scRNA-seq data was aligned to the Nipponbare reference genomes [29], respectively, and counted using the Cell Ranger pipelines (version 2.0, 10 × Genomics), resulting in 39,219 genes. The dataset has six different cell subpopulations, which are Root hair (121), Epidermis (1000), Stele (1000), Cortex (1000), Epidermis (NRH) Near_root (131), Endodermis (211). To benefit the model evaluation, the dataset used in this research was split into training and test datasets according to 7:3. More dataset details are provided in the Supporting Information (Additional file 2: Table S1). The Python packages, Numpy (version 1.21.6), Pandas (version1.3.5) and Scanpy (version 1.9.1) were used to read and process the data.

In addition, we constructed an *Arabidopsis* root tips scRNA-seq dataset containing a total of 4130 cells, and available from the National Center for Biotechnology Information (GSE152766) [30]. Based on the same strategy, the scRNA-seq data were aligned to an *Arabidopsis* genome BSgenome object (“BSgenio.Athaliana.TAIR.TAIR9”) with an annotation file for the TAIR10 gene and counted using the Cell Ranger pipelines, resulting in 25,261 genes [30]. The dataset, including six different cell subpopulations, Endodermis (546), Lateral Root Cap (832), Atrichoblast (564), Trichoblast (721), Cortex (696) and Procambium (771), is split into a 70% training dataset and a 30% test dataset. More dataset details are provided in the Supporting Information (Additional file 2: Table S2).

Bulk RNA-seq data of rice root tissues were collected from the National Center for Biotechnology Information (PRJNA639386) [31]. We used root and Nipponbare as parameters to filter in the PPRD database and combined publication time and academic impact to select PRJNA639386 for further study. Yu et al. [25] described dataset processing, which included raw read alignments to Nipponbare reference genomes (Os-Nipponbare reference IRGSP-1.0) using HISAT2 (version 2.1.0) with parameters (“-max-intron-length = 20,000 -k 1 -dta –n-ceil -L,0,0.15”) and removed duplicated reads using SAMtools rmdup (version 1.4.1) (See references for more details). The data contain transcriptional expression profiles of root tissues of Nipponbare seedlings collected at 0.5 h and 48 h after NaCl (140 mm) treatment. Six uniform seedlings were selected for each group.

### F-score

F-score is a simple and effective feature selection method, which estimates the weight of each feature by detecting the balance between quantity and quality, to eliminate redundant and noisy information contained in features [32, 33].

1 $$\begin{array}{c}{F}_{i}=\frac{{\left({\overline{x} }_{i}^{\left(+\right)}-{\overline{x} }_{i}\right)}^{2}+{\left({\overline{x} }_{i}^{\left(-\right)}-{\overline{x} }_{i}\right)}^{2}}{\frac{1}{{n}_{+}-1}\sum_{k=1}^{n+}{\left({\overline{x} }_{k,i}^{\left(+\right)}-{\overline{x} }_{i}^{\left(+\right)}\right)}^{2}+\frac{1}{n-1}\sum_{k=1}^{n-}{\left({x}_{k,i}^{\left(-1\right)}-{\overline{x} }_{i}^{\left(-\right)}\right)}^{2}}\end{array}$$

where $\overline{{x }_{i}}$ represents the average of the $i$ th feature of the whole. ${\overline{x} }_{i}^{\left(+\right)}$ is the number of positive samples, ${\overline{x} }_{i}^{\left(-\right)}$ is the number of negative samples. ${\overline{x} }_{k,i}^{\left(+\right)}, {\overline{x} }_{i,i}^{\left(-\right)}$ are the $i$-th feature of the $k$-th positive and negative instances, respectively. The larger the F-score value, the stronger the distinguishing degree of the feature among different categories.

### MIC

The core idea of MIC is: if there is a relationship between two variables, there will be a grid that can split the scatter graph of the two variables to encapsulate this relationship, and then normalize these mutual information values to ensure a fair comparison between grids of different dimensions [34–36]

2 $$I\left( {X;Y} \right) = \mathop \sum \limits_{{x,y}} p\left( {x,y} \right)log\frac{{p\left( {x,y} \right)}}{{p\left( x \right)p\left( y \right)}} = H\left( X \right) - H(X|Y)~~~$$

where $I\left(X;Y\right)$ representing Mutual Information Entropy, is a measure of the information about variable $X$ (or $Y$) contained in variable $Y$ (or $X$).

### Pseudobulk differential expression analysis

Recent studies have demonstrated the superior performance of pseudobulk differential expression analysis in single-cell RNA-sequencing analysis [37]. Bioconductor's SingleCellExperiment class was used to store single-cell assay data [38]. Differential expression analysis was performed on the scRNA-seq data (3463 cells, 39,217 genes) using DESeq2 [39], and shrinkage estimation was applied to the dispersion and fold change to improve the stability and interpretability of the estimates (log2Foldchange > 2 or < − 2, P value < 0.05, and pAdjustMethod = “BH”). The DESeq2 package is available at http://www.bioconductor.org/packages/release/bioc/html/DESeq2.html.

Mean gene expression profiles in different cell subpopulations were analyzed using a scRNA-seq dataset of Nipponbare root tips. The raw read count was first normalized (Log2(count + 1)), and then the data was mean valued. The Python package Matplotlib (version 3.5.3) was used to plot boxplots.

### Biological analysis and visualization

We conducted a more comprehensive analysis and evaluation of the predictive power of the 110 marker genes in cell subpopulation identification. The Clustering analysis software implemented in Scanpy (version 1.9.1) was used to determine specific cell subpopulations of marker genes, with all parameters selected by default. Using Pandas (version 1.4.4), perform Pearson correlation analysis on six rice root cell populations at the level of 110 marker genes. PAGA analysis was performed using Scanpy, and UMAP visualization utilized the python package umap-learn version 0.3.9, with all parameters set to default. Specifically, cell trajectory analysis was performed using PAGA implemented on Scanpy for both the original feature dataset and the dataset with only 110 genes, with default parameters. We used the enrichKEGG function in the clusterProfiler package (version 4.6.2) to perform functional enrichment analysis. We employed the “org.Osativa.eg.db” software package (https://github.com/xuzhougeng/org.Osativa.eg.db) to facilitate the conversion between MSU and RAP-DB.

### Model construction of NRTPredictor

Nipponbare root cell subpopulation gene expression profiles were used as input features to train the machine learning model. In exploratory data analysis, essential relationships and weights between features can be used to filter out weaker or less interesting information. MIC [34–36], CV2 [40], and F-score [41, 42] were used to score and rank the weights of each gene in the training model, respectively. The genes with weight scores less than or equal to zero were also removed.

The incremental feature selection (IFS) [43, 44] was applied to train XGBoost [45], SVM [46], Lightgbm [47], and RFC [48] base models, and 110 optimal genes were identified by comparing their predictive performance. The ensemble methods in MLxtend cover the majority of voting, stacking, and stacked generalization. Based on the 110 optimal gene features and model performance, the above four models were integrated to construct the ensemble model (NRTPredictor) through the weight voting strategy. Regarding the estimation of the voting weights, we determined the weights based on the predictive performance of each individual model (SVM: 1.0, RFC: 1.0, Lightgbm: 1.0 and XGBoost: 1:0). MLxtend is available at https://github.com/rasbt/mlxtend.

### Model evaluation

The four classic metrics were used to quantify the performance of model predictions, including Accuracy (Acc), Recall (Re), Precision (Pre), and F1 measure (F1), defined as [49–54]:

3 $$\mathrm{Accuracy}=\frac{TP+TN}{TP+TN+FP+FN}$$

4 $$\mathrm{Recall}=\frac{TP}{TP+FN}$$

5 $$\mathrm{Precision}=\frac{TP}{TP+FP}$$

6 $${\text{F}}1{\text{~measure}} = \frac{{2*\left( {precision*recall} \right)}}{{precision + recall}}$$

where $TP, TN, FP \mathrm{and} FN$ represent the numbers of true positives, true negatives, false positives and false negatives, respectively. In addition, ROC was used to evaluate the performance of the NRTPredictor [55].

### Supplementary Information

**Additional file 1: Figure S1.** The workflow of constructing NRTPredictor. **Figure S2.** Comparative Venn diagram of the top 110 genes of MIC and 1216 genes of Pseudobulk. **Figure S3. **UMAP shows potential marker genes for rice root cell fate determination. **Figure S4.** Comparison of marker genes selected by MIC_SVM using split violin plots. The expression level of marker genes in specific cells is shown on the left (Blue), and the total expression level in the remaining five cell types is shown on the right (Orange). **Figure S5.** Expression levels of 12 genes in different tissues. **Figure S6.** Association of cell subpopulations with different stress conditions. Small circles represent genes and marker cell subpopulations, large circles represent stress states. Based on the PPRD database, we obtained RNA-seq data statistics for rice under stress conditions when searching for the keywords “Nipponbare” and “root tips”. Subsequently, we annotated the 12 genes we unearthed to understand their associations with different cell subpopulations and their relationships with various stress conditions. **Figure S7** Transcript levels of root tissues collected from Nipponbare seedlings treated with NaCl for 0.5 h and 48 h.

**Additional file 2: Table S1.** The cell subpopulations of Nipponbare root tips data composition. **Table S2.** The cell subpopulations of Arabidopsis root tips data composition. **Table S3.** Performance comparison between NRTPredictor and the other algorithms (Arabidopsis dataset). **Table S4.** Pseudobulk differential expression analysis of rice root scRNA-seq data (3463 cells，39,217 genes) using DESeq2. **Table S5.** Marker genes and cell states. **Table S6.** Results of Kyoto Encyclopedia of Genomes (KEGG) enrichment analysis of 110 marker genes. **Table S7.** Annotation form for 110 marker genes.

## Data Availability

All data generated or analyzed during this study are included in this published article.

## Abbreviations

scRNA-seq: Single-cell transcriptome
XGBoost: EXtreme Gradient Boosting
MIC: Maximal information coefficient
IFS: Incremental feature selection
SVM: Support vector machine
RFC: Random Forest Classifier
UMAP: Uniform Manifold Approximation and Projection
PAGA: Partition-based graphical abstraction
KEGG: Kyoto Encyclopedia of Genes and Genomes
ROC: The receiver operating characteristic curve
MIC_SVM: MIC combined with SVM
