# Supplementary material for: NRTPredictor: identifying rice root cell state in single-cell RNA-seq via ensemble learning
Source: Plant Methods. 2023 Nov 4;19:119. doi: 10.1186/s13007-023-01092-0 (PMC10625708; doi:10.1186/s13007-023-01092-0)
Supplement: Supplementary file 1 — Additional file 1: Figure S1. The workflow of constructing NRTPredictor. Figure S2. Comparative Venn diagram of the top 110 genes of MIC and 1216 genes of Pseudobulk. Figure S3. UMAP shows potential marker genes for rice root cell fate determination. Figure S4. Comparison of marker genes selected by MIC_SVM using split violin plots. The expression level of marker genes in specific cells is shown on the left (Blue), and the total expression level in the remaining five cell types is shown on the right (Orange). Figure S5. Expression levels of 12 genes in different tissues. Figure S6. Association of cell subpopulations with different stress conditions. Small circles represent genes and marker cell subpopulations, large circles represent stress states. Based on the PPRD database, we obtained RNA-seq data statistics for rice under stress conditions when searching for the keywords “Nipponbare” and “root tips”. Subsequently, we annotated the 12 genes we unearthed to understand their associations with different cell subpopulations and their relationships with various stress conditions. Figure S7 Transcript levels of root tissues collected from Nipponbare seedlings treated with NaCl for 0.5 h and 48 h. [file 13007_2023_1092_MOESM1_ESM.docx]

**NRTPredictor: identifying rice root cell stages single-cell RNA-seq via ensemble learning**

Hao Wang^†^, Yu-Nan Lin^†^, Shen Yan^†^, Jing-Peng Hong, Jia-Rui Tan, Yan-Qing Chen*, Yong-Sheng Cao*, Wei Fang*

The Innovation Team of Crop Germplasm Resources Preservation and Information, Institute of Crop Sciences, Chinese Academy of Agricultural Sciences, Beijing 100081, China.

**^†^**These authors contributed equally to this work.

* Corresponding authors: Wei Fang, The Innovation Team of Crop Germplasm Resources Preservation and Information, Institute of Crop Science, Chinese Academy of Agricultural Sciences, Beijing 100081, China, E-mail: fangwei@caas.cn; Yong-Sheng Cao, The Innovation Team of Crop Germplasm Resources Preservation and Information, Institute of Crop Science, Chinese Academy of Agricultural Sciences, Beijing 100081, China, E-mail: caoyongsheng@caas.cn; Yan-Qing Chen, The Innovation Team of Crop Germplasm Resources Preservation and Information, Institute of Crop Science, Chinese Academy of Agricultural Sciences, Beijing 100081, China, E-mail: chenyanqing@caas.cn.


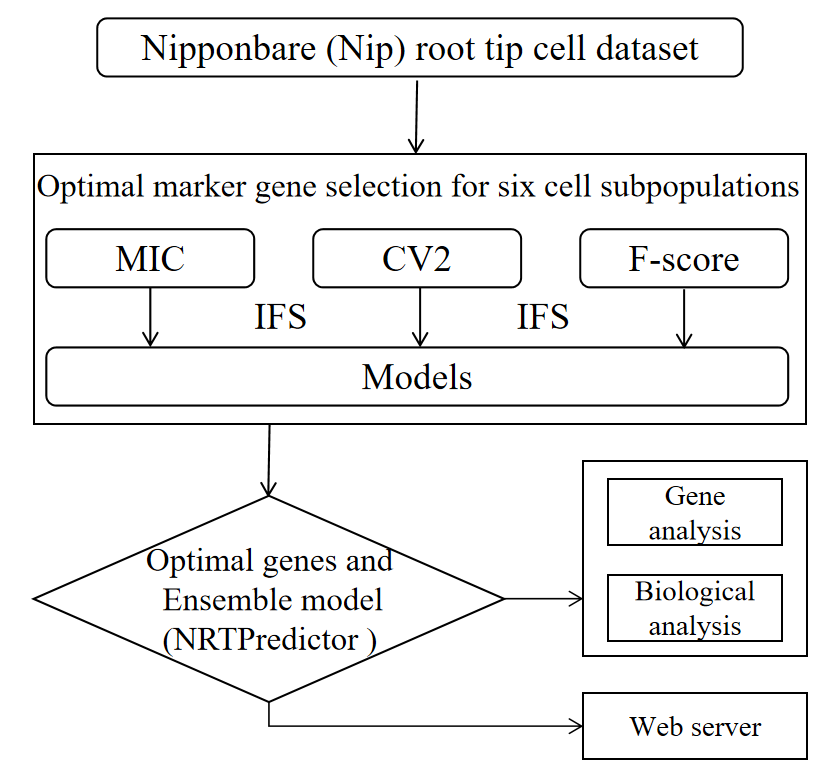


**Supplementary Figure 1** The workflow of constructing NRTPredictor.


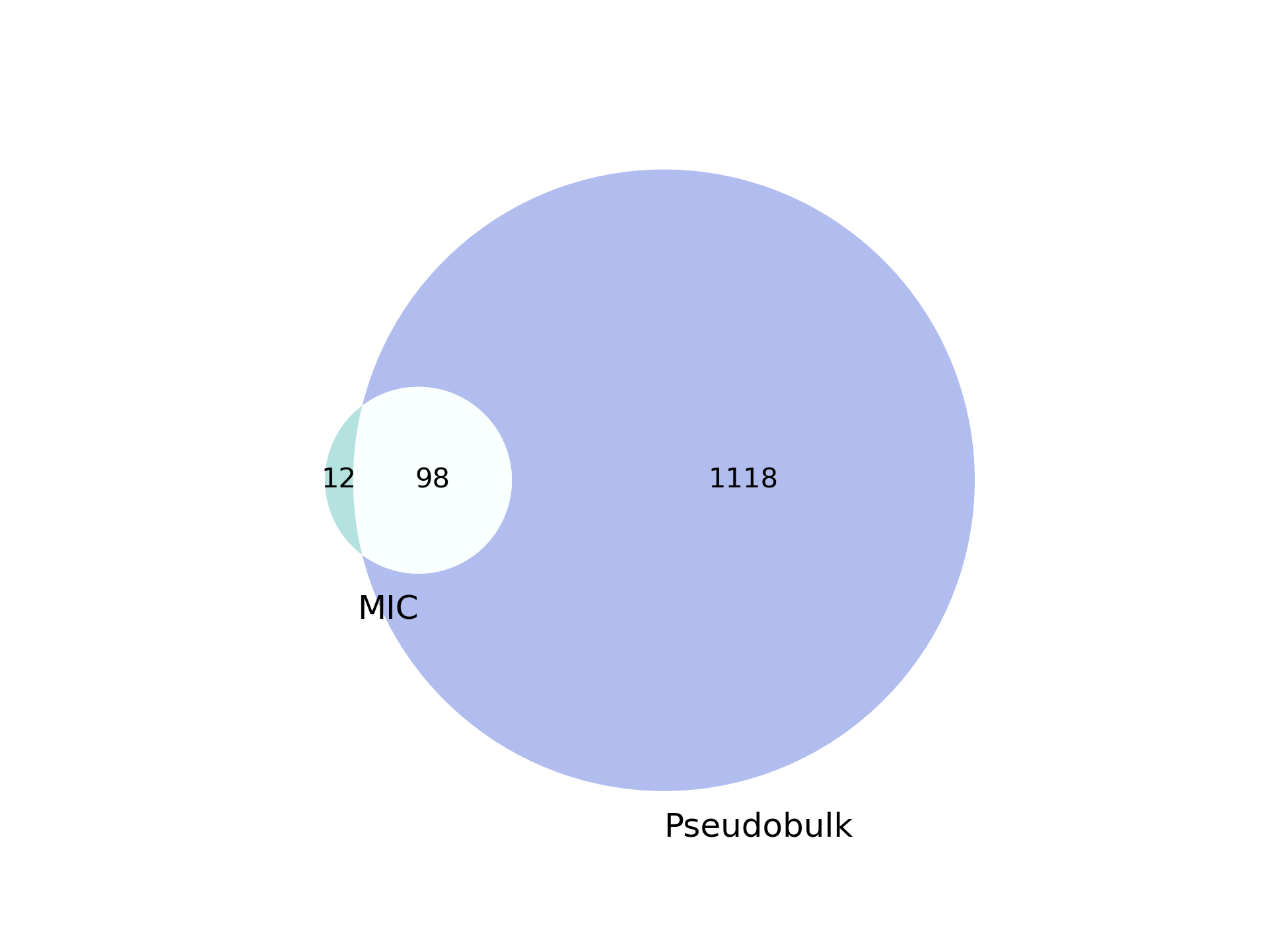


**Supplementary Figure 2** Comparative Venn diagram of the top 110 genes of MIC and 1,216 genes of Pseudobulk.


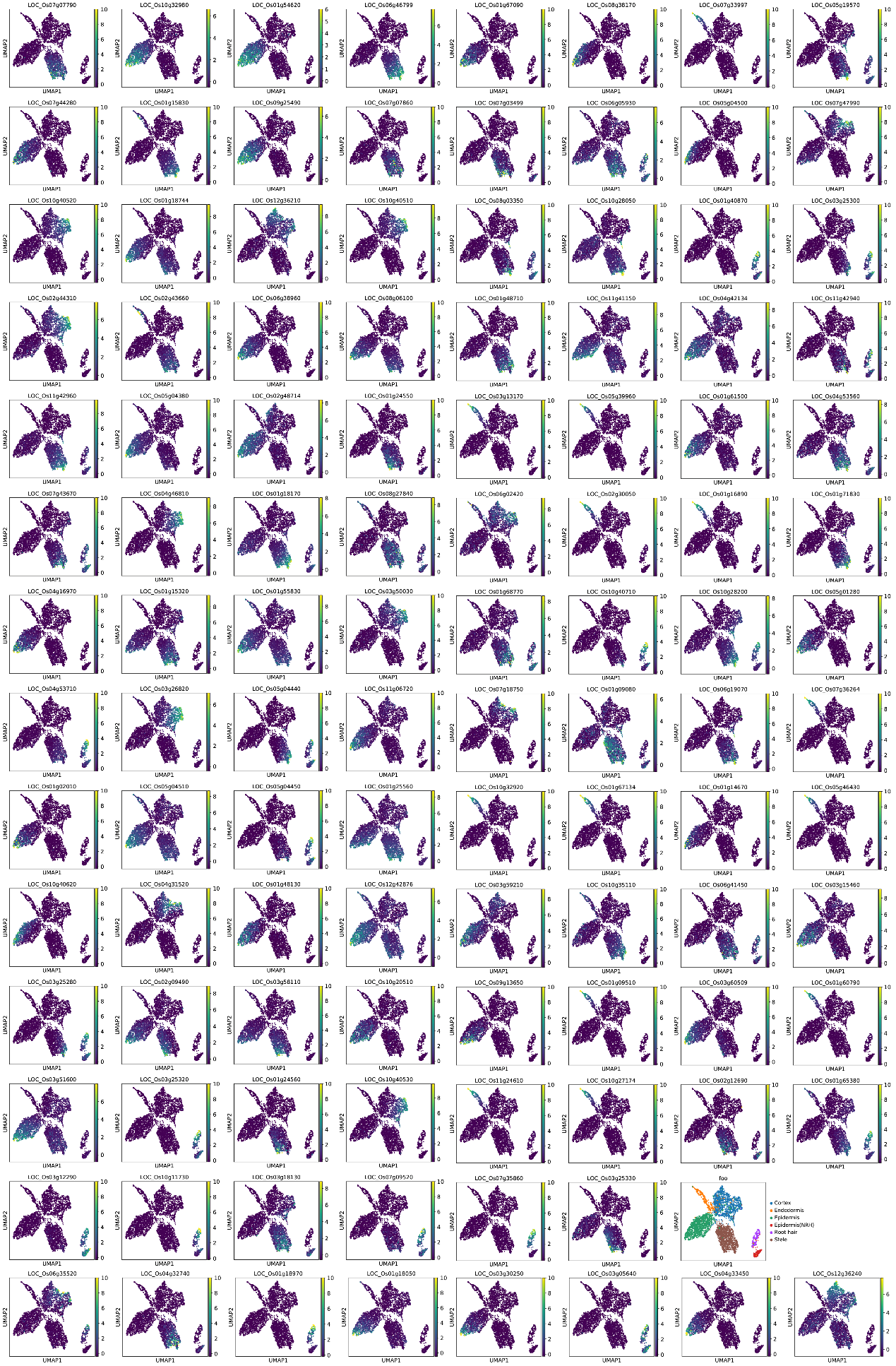


**Supplementary Figure 3** UMAP shows potential marker genes for rice root cell fate determination.


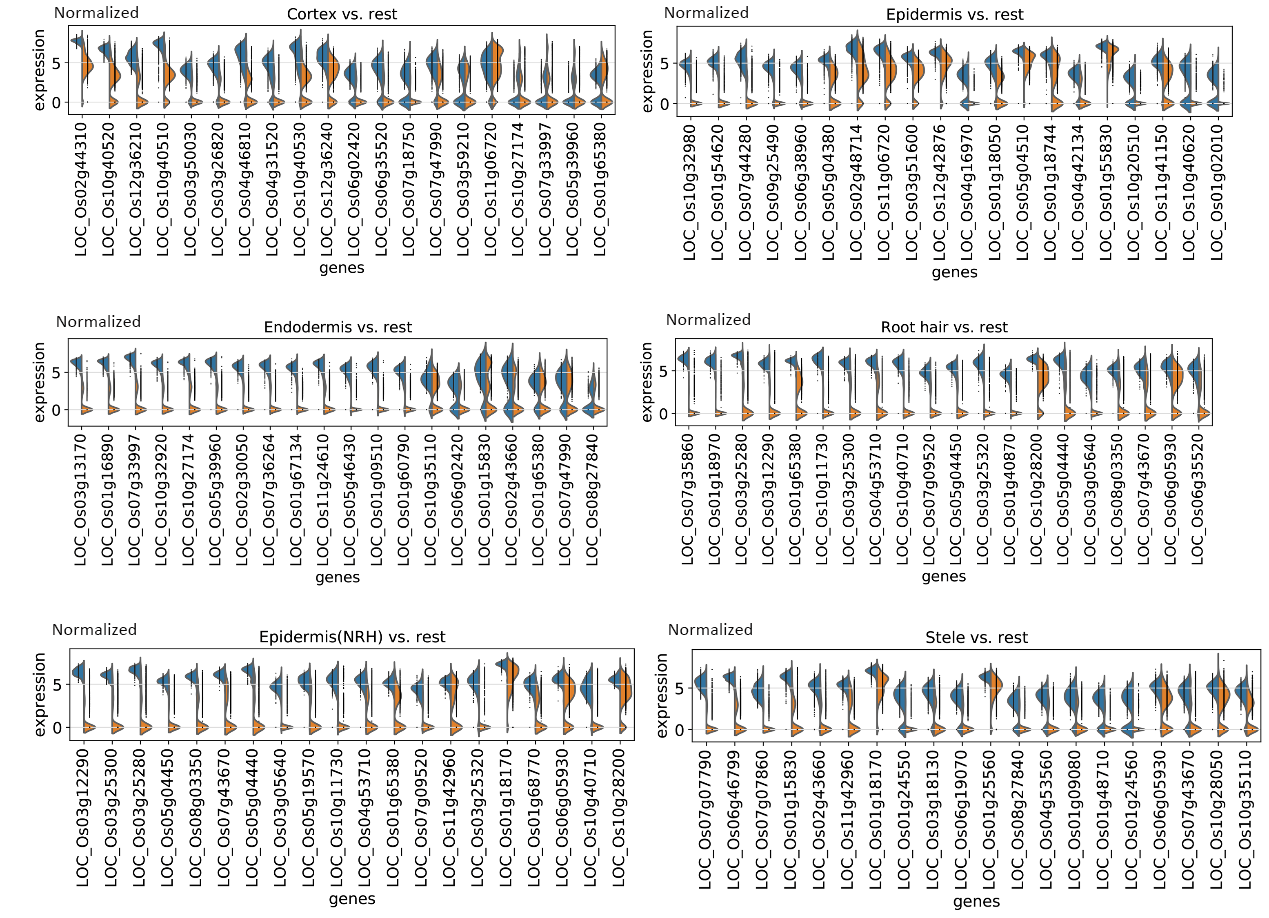
s

**Supplementary Figure 4** Comparison of marker genes selected by MIC_SVM using split violin plots. The expression level of marker genes in specific cells is shown on the left (Blue), and the total expression level in the remaining five cell types is shown on the right (Orange).


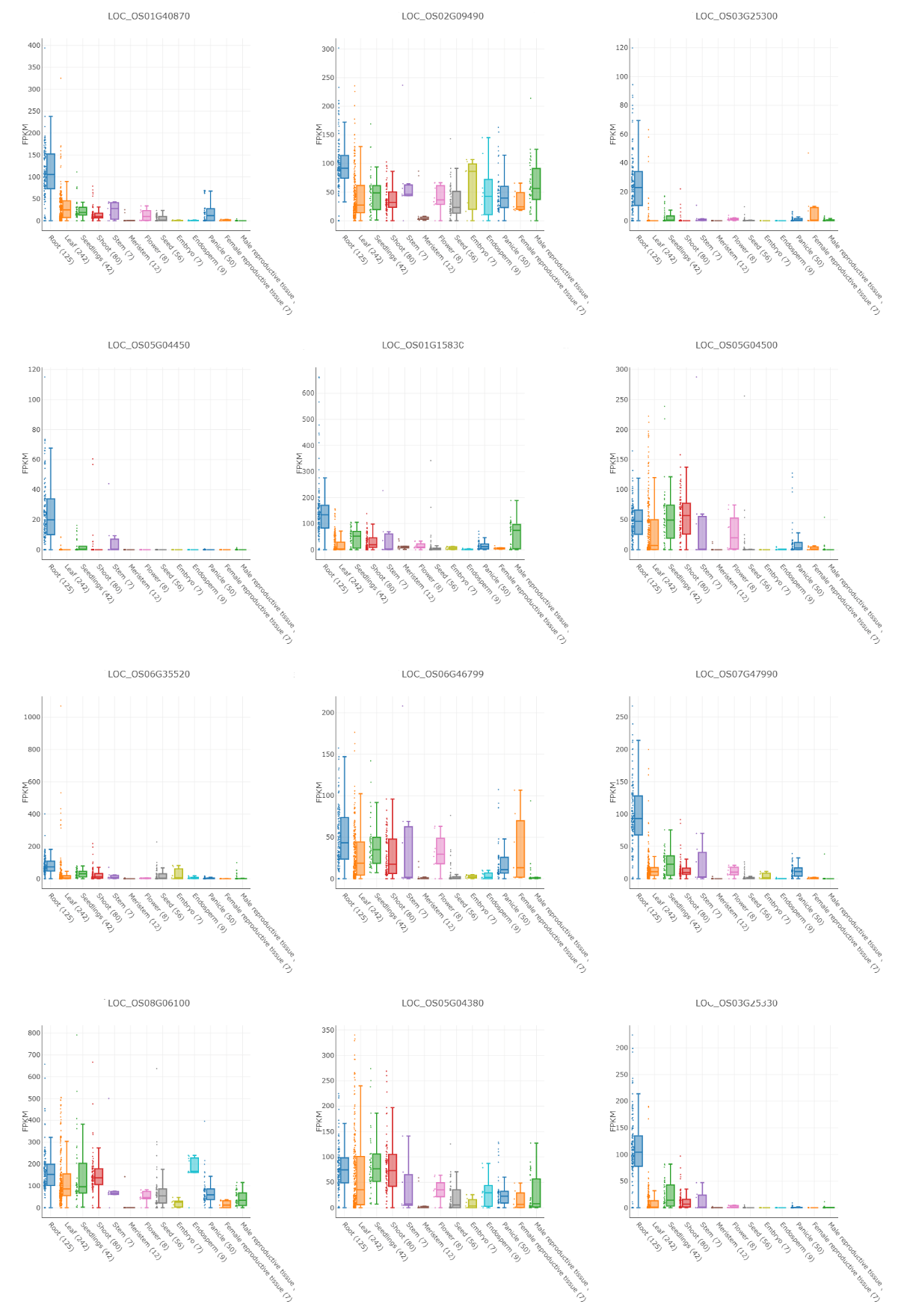
.

**Supplementary Figure 5** Expression levels of 12 genes in different tissues.


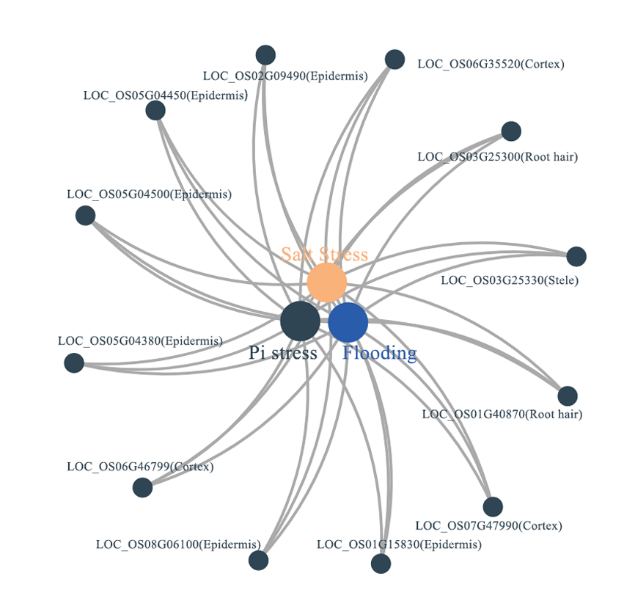


**Supplementary Figure 6** Association of cell subpopulations with different stress conditions. Small circles represent genes and marker cell subpopulations, large circles represent stress states. Based on the PPRD database, we obtained RNA-seq data statistics for rice under stress conditions when searching for the keywords "Nipponbare" and "root tips". Subsequently, we annotated the 12 genes we unearthed to understand their associations with different cell subpopulations and their relationships with various stress conditions.


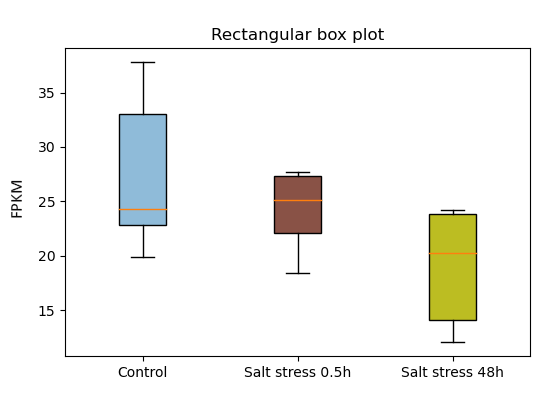


**Supplementary Figure 7** Transcript levels of root tissues collected from Nipponbare seedlings treated with NaCl for 0.5 h and 48 h.
